# Impact of nebulizers on nanoparticles-based gene delivery efficiency: *in vitro* and *in vivo* comparison of jet and mesh nebulizers using branched-polyethyleneimine

**DOI:** 10.1080/10717544.2025.2463428

**Published:** 2025-02-10

**Authors:** Rosy Ghanem, Xavier Buin, Tanguy Haute, Justine Philippe, Ghalia Kaouane, Lara Leclerc, Maël Guivarch, Tony Le Gall, Jérémie Pourchez, Tristan Montier

**Affiliations:** a Univ Brest, Inserm, EFS, UMR 1078, GGB, Brest, France; b CHU de Brest, Service de Génétique Médicale et de Biologie de la Reproduction, Brest, France; c Mines Saint-Etienne, Univ Lyon, Univ Jean Monnet, INSERM, U 1059 Sainbiose, Centre CIS, Saint-Etienne, France

**Keywords:** Gene delivery, aerosol, nebulization, branched-polyethyleneimine, lung, jet nebulizer, mesh nebulizer, cystic fibrosis

## Abstract

Nanoparticles-based gene delivery has emerged as a promising approach for the treatment of genetic diseases based on efficient delivery systems for therapeutic nucleic acids (NAs) into the target cells. For pulmonary diseases such as cystic fibrosis (CF), chronic obstructive pulmonary diseases (COPD), infectious disease or lung cancer, aerosol delivery is the best choice to locally deliver NAs into the lungs. It is, therefore, important to investigate the effects of nebulization conditions on the efficiency of delivery. To this purpose, the non-viral vector branched polyethyleneimine (b-PEI, 25 kDa) was investigated for plasmid delivery by aerosol. Two types of nebulizers, jet nebulizer and mesh nebulizer, were compared regarding the properties of the nanoparticles (NPs) formed, the efficiency of NAs delivery *in vitro* and *in vivo* models and the pulmonary deposition. The results indicate that the mesh nebulizer has a better gene delivery performance than the jet nebulizer in this application. This superiority was demonstrated in terms of size, concentration, distribution of NPs and efficiency of NAs delivery. However, pulmonary deposition appears to be similar regardless of the nebulizer used, and the difference between the two systems lies in the inhalable dose. These results underline the crucial role of nebulization techniques in optimizing aerosol-mediated gene delivery by b-PEI and highlight the potential of mesh nebulizers as promising tools to improved gene therapy. Therefore, the comparison must be performed for each gene therapy formulation to determine the most suitable nebulizer.

## 1. Introduction

Aerosol therapies are a quick, noninvasive, and easy way to reach the lungs. This type of delivery is perfect for the localized administration of medication into the lungs and can be used for various diseases such as cystic fibrosis (CF), asthma, infectious diseases, lung cancer or chronic obstructive pulmonary disease (COPD) (Anderson et al., 2022). This administration method is often used to deliver anesthetics, bronchodilators, antibiotics or mucoactive drugs directly into the airways (Dennis et al., 1990; Barjaktarevic and Milstone, 2020; Hocquigny et al., 2024). Additionally, aerosol delivery is a technique that avoids the hepatic first-pass effect, thereby limiting side effects and increasing the bioavailability of the drug in the pulmonary tract (Douafer et al., 2020). There are various options for the administration of aerosols: inhalers or nebulizers (Peng et al., 2024). While inhalers are used to administer solid or liquid medications, nebulizers can only administer liquid formulations. This is because the nebulization process is based on the conversion of a liquid solution into microdroplets that form the inhalable mist (Martin and Finlay, 2015). Different types of nebulizers have been developed over time. The first type, for example, was the jet nebulizer, which uses gas, e.g. compressed air, to generate the aerosol. Jet nebulizers operate based on the Venturi effect and the Bernoulli principle and were originally developed for drugs that are not suitable for inhalers, such as antibiotics (Tobramycin) or recombinant proteins such as Dornase alpha (Pulmozyme) (Fiel, 1995; Shire, 1996; Ari, 2014; Anderson et al., 2022). The mesh nebulizer is another type of nebulizer in which the microdroplets are generated by the vibration of a mesh with micro-openings. This type of nebulizer represents a new generation of devices that extrude the liquid solution through thousands of micropores (Fossat et al., 2022). Depending on the drug and formulation, the choice of nebulizer must be carefully considered to increase the exposure dose and maintain the physicochemical integrity of the drug (Watts et al., 2008). In addition to conventional drugs, nebulization is a good alternative to systemic administration for innovative treatments such as gene delivery. Indeed, lung gene therapy is suitable for nebulization as it directly reaches the target cells that form the pulmonary epithelium (Kassab et al., 2023). Different approaches can be used for gene delivery including viral and non-viral strategies. Although viral gene therapy is more efficient in terms of efficacy and is already used in clinics for various diseases such as spinal muscular atrophy (SMA) type I (Zolgensma), non-viral vectors have the advantage of triggering the immune system less strongly and can, therefore, be re-administered (Lindberg et al., 2015; Butt et al., 2022; Lundstrom, 2023; Ogbonmide et al., 2023; Wang et al., 2023). Non-viral strategies can also compact longer nucleic acids (NAs) constructs than viral vectors, allowing for the supplement of full-length transgenes such as CFTR (Cystic Fibrosis Transmembrane conductance Regulator). Various vehicles have been developed for non-viral delivery and efficient transport of NAs. For example, the cationic polymer polyethyleneimine (PEI) has been extensively investigated for NAs delivery. PEI can be linear (l-PEI) with secondary amines or branched (b-PEI) composed of primary, secondary and tertiary amines (Wagner et al., 2013). However, b-PEI has been shown to be more efficient by aerosol delivery, as demonstrated in several publications (Gautam et al., 2000; 2001; Davies et al., 2008). Indeed, b-PEI can (i) well condense pDNA through electrostatic interactions, (ii) form stable complexes with NAs, (iii) support the shear forces of the jet nebulizer and (iv) facilitate cellular uptake (Densmore et al., 2000; Godbey et al., 2000; Rudolph et al., 2002). The better compromise between efficiency and cell toxicity is found with 25 kDa b-PEI (Godbey et al., 1999; Sundaram et al., 2007). This latter has been used to transfect mice and ovine lungs and has already demonstrated its efficiency in transfecting ciliated epithelia (Gautam et al., 2000; Rudolph et al., 2005; McLachlan et al., 2011). However, the performance of b-PEI-mediated gene delivery *via* aerosolization is influenced by several factors, including the choice of nebulization technique. Therefore, in this study, two nebulizers, (i) jet nebulizer and (ii) mesh nebulizer, were compared using nanoparticles (NPs) consisting of b-PEI and a CpG-free plasmid to determine which one is best suited for pulmonary gene transfer applications. This comparison was performed in terms of NPs characterization, gene transfection efficiency *in vitro* and *in vivo* and pulmonary deposition.

## 2. Material and methods

### 2.1. pDNA production and characterization

The plasmid used for the experiment was a CpG-free plasmid encoding the luciferase gene, pGM144 (3.7 kb) (Hyde et al., 2008). This latter was amplified in GT115 *Escherichia coli and* then extracted using a NucleoBond PC 10,000 purification kit (Macherey-Nagel) according to the manufacturer's protocol. All plasmid productions were quantified using nanodrop (NanoDrop ND-1000 Spectrophotometer). The ratio 260/280 nm was also evaluated and only the plasmids with a ratio between 1.8 and 2.2 were used for the experiments. To characterize plasmids production, double-digestion restriction enzymatic gel was conducted using EcoRI/KpnI or BamHI/XbaI.

### 2.2. Nanoparticles (NPs) preparation

Branched-polyethyleneimine (b-PEI, 25 kDa, Sigma-Aldrich) was dissolved in HEPES solution (20 mM) to prepare a stock solution of b-PEI at 20 mg/mL. Complexation was performed in sterile water (Versylene^®^ Fresenius) at room temperature with pGM144 (250 µg or 2 mg depending on the experiment) and b-PEI at N/P 40 (nitrogen/phosphate ratio). The solution of b-PEI was mixed volume to volume with the solution of pGM144 and then allowed to rest at room temperature for 30 min before being used for experiments.

### 2.3. Nanosizer and nanoparticle tracking analysis before and after nebulization

The NPs hydrodynamic diameter as well as the zeta potential were measured using Zetasizer Nano ZS (Malvern Panalytical). NPs before nebulization were diluted in sterile water (1/10) before measurements. To collect NPs after nebulization a 96-well plate containing 200 µL of sterile water per well was exposed to the aerosol. Then, NPs were collected and analyzed using Zetasizer without further dilution. The sample was also proceeded on the Nanoparticle Tracking Analysis (NTA), NS300 (Malvern Panalytical) to assay the size and concentration of the sample. This latter was determined based on the theoretical volume of aerosolized solution deposed per well (26 µL per well for 2.5 mL nebulized).

### 2.4. DNA integrity via electrophoresis

To assay pDNA integrity after nebulization, an electrophoresis was conducted. Briefly, 10 µL of NPs before and after aerosol were incubated either with dextran sulfate (20 mg/mL) or sterile water for 30 min at room temperature. Dextran sulfate releases NAs *via* electrostatic competition. Electrophoresis was then performed in 0.8% agarose gel stained with ethidium bromide (Biorad) in mupid^®^-One at 100 V for 30 min. The gel was visualized using a UV transilluminator (Uvitec Essential V6).

### 2.5. Cell culture

Four cell lines were used in this study. A549 (ATCC CCL-185) and Calu-3 (ATCC HTB-55) are two human adenocarcinoma cell lines. They were cultivated in Dulbecco’s Modified Eagle Medium (DMEM) supplemented with 10% heat-inactivated Fetal Bovine Serum (FBS), 1% antibiotics (10,000 U.mL^−1^ Penicillin, 10,000 µg.mL^−1^ Streptomycin) and an additional 1% of L-Glutamine. 16HBE14o- (kindly provided by DC Gruenert) (Cozens et al., 1994) and CFBE41o- (mutated homozygote p.Phe508del) (Merk SCC151) are immortalized human bronchial epithelial cells and were maintained in Eagle’s Minimal Essential Medium (EMEM) supplemented with 10% heat-inactivated Fetal Bovine Serum (FBS), 1% antibiotics (10,000 U.mL^−1^ Penicillin, 10,000 µg.mL^−1^ Streptomycin) and an additional 1% of L-Glutamine. All cells were maintained at 37 °C in a humidified atmosphere containing 5% CO_2_. All cell lines were routinely tested to ensure they were free from mycoplasma contamination.

### 2.6. Exposure box conception and validation assays

To expose cell culture to aerosol or collect NPs after nebulization an exposure box was used. This latter was designed using “123D design software” and 3D printed in poly lactic acid. Different shapes and sizes have been tested, and the better compromise has been settled to a pyramid 150 mm high (∼1.1 m^3^) (Figure S1). The Luciferase reporter gene has been used to determine the repartition of aerosol inside the box, and the experiment was repeated 5 times. Results have been rescaled with a min–max normalization and represented in a heat map. Boxplot of the distribution of values have also been performed across three predefined spatial zones (i.e., Center, Middle, and Periphery). Zones were defined based on the distances of each square i from the center, where each square is considered of side 1: ${\text{Zone}}_{i}\{\begin{array}{c} \text{Center},\;\text{if}\;d_{i}<2\;\\ \text{Middle},\;\text{if}\;2<d_{i}<5\;\\ \text{Periphery},\;\text{if}\;d_{i}\geq 5\; \end{array}$

### 2.7. In vitro aerosol assays

Aerosol assays on *in vitro* cells were conducted either on immerged cells cultivated in 96-well plates or air-liquid interface (ALI) cells. For immerged cells, A549 and CFBE41o- were seeded at a density of 20,000 cells per well, and Calu-3 and 16HBE14o- at a density of 40,000 cells per well. The following day, cells were exposed to the aerosol in the 150 mm high pyramidal exposure box. ALI cultures were performed on 24-well transwells (0.4 µm pore diameter, Sarstedt) with 16HBE14o- cells as previously described (Ghanem et al., 2023). Cells were seeded at a density of 150,000 cells per transwell and cultivated under liquid conditions for 1 week. The upper medium was then removed to mimic airway epithelial conditions. The bottom medium was changed every 2 to 3 days. After 3 weeks of ALI culture, cells were exposed to the aerosol as previously mentioned. Nebulization was performed with a jet nebulizer (PARI LC Plus) or a mesh nebulizer (PARI eFlow). The volume of nebulization was fixed at 2.5 mL for both nebulizers and NPs were loaded with 250 µg of pGM144 for immerged cells. However, for nebulization on ALI cells, the volume used was 5 mL with 2 mg of pGM144 to stay in the same condition as *in vivo* and aerosol characterization (NGI/GTI) experiments (see below).

### 2.8. Luciferase activity and viability assays

Twenty-four hours after nebulization, cells were lysed with 75 µL of passive lysis buffer 0.5× (PLB, Promega) for 96-well plate (immerged cells) or 200 µL of PLB 0.5× for ALI culture (24-well transwells). Luciferase expression was quantified using Luciferase Assay System (Promega) according to the manufacturer protocol. A multimode microplate reader (Mithras^2^ LB943, Berthold) was used to inject 25 µL of luciferin to an equal volume of cell lysate and to measured luminescence. Another 25 µL of cellular lysate were used for proteins quantification using BC Assay kit (Interchim). Briefly, 200 µL of BCA reagent were added to 25 µL of the sample and incubated at 37 °C for 30 mins prior to read absorbance at 540 nm. To determine the sample concentration, a standard curve was performed using bovine serum albumin. Results are reported in Relative Light Unit per milligram of total proteins (RLU/mg of proteins). Cellular viability was measured using ViaLight Plus Cell Proliferation and Cytotoxicity BioAssay kit (Lonza). The reagent was reconstituted following manufacturer protocols and 50 µL was added in 25 µL of cellular lysate. After 2 min of incubation, the luminescence was measured using a multimode microplate reader (Mithras^2^ LB943, Berthold). Results are expressed in percentage of viability of non-exposed cells used as reference (100%).

### 2.9. In vivo experiments

All *in vivo* experiments were approved under protocols APAFIS#40468 by the CEEA 74 (Animal Experimentation Ethics Committee Number 74) and the MESRI (Ministry of Higher Education, Research, and Innovation). The animal experiments were conducted following ARRIVE guidelines. Experiments were conducted on ten Balb/cAnNRj mice assigned to either the jet or mesh nebulizer group. The mice were provided by JanvierLabs at 5 weeks old. After 1 week of acclimation, the mice were trained to enter to the contention tube in 4 min increments per day. The maximum time of mice contention was 28 min. One week later, nebulization was conducted in two groups of five mice, one group received treatment through jet nebulizer and the other group through mesh nebulizer (Figure S2). The volume of the nebulization was fixed at 5 mL with NPs prepared with b-PEI and 2 mg of pGM144 at N/P40. Mice were then sacrificed one week later, and their tracheas and lungs were harvested. Before sacrifice, the mice were anesthetized with a ketamine/xylazine ratio of 10/1 (at a dose of 100 mg/kg body weight of ketamine and 10 mg/kg body weight of xylazine) *via* the intraperitoneal route to perform intracardiac puncture, and then euthanized by cervical dislocation. The organs were then crushed in 1 mL of passive lysis buffer 0.5x (PLB, Promega) using FastPrep FP120 sample disrupter (Thermo Electron) and centrifuged at 10 000 rpm for 10 mins. Then, the supernatant was used to perform luciferase assay and proteins quantification as describe before. Results are reported in Relative Light Unit per milligram of total proteins (RLU/mg of proteins).

### 2.10. Next-generation impactor assay

An NGI (Next Generation Impactor, Copley Scientific of Nottingham, UK) is a cascade impactor used to evaluate the properties of the aerodynamic distribution of the aerosol such as MMAD (mass median aerodynamic diameter) and GSD (geometric standard deviation). The separation of the particles depends on the velocity and aerodynamic particle size. The NGI is composed of eight nozzle pieces, corresponding to seven size-fractionation stages and a micro-orifice collector (MOC) that takes the place of a final filter. The airflow passes through the device while allowing the particles to move and reach the trays, where they acquire the associated cutoff diameter. The setup consisted of an NGI connected to a vacuum pump (High Capacity Pump Model HCP5, Copley Scientific of Nottingham, UK) at a flow rate of 15 L.min^−1^ (particle size range from 0.98 to 14.10 microns). The experiment consisted of delivering NPs (b-PEI with 2 mg of pGM144 at N/P40) aerosol (5 mL loaded) with the nebulizers into the opening of the NGI until the nebulizer was deactivated. After the experiment, the different fractions were collected with 2 mL of sterile water and stored at +4 °C prior to NPs content analyses. MMAD and GSD of nebulized particles were determined by drawing the cumulative curve of mass *vs* size.

### 2.11. Glass twin impinger assay

A Glass Twin Impinger (GTI, custom-made glassware made by Verrerie de Laboratoire CHENU, Issoire, France) is a two-chamber glass device that mimics the airways from the throat to the lung. It was used to separate the aerosol respirable dose (lower impingement) from the non-respirable dose (upper impingement). The set up consisted of a GTI connected to a vacuum pump (Low-Capacity Pump Model LCP5, Copley Scientific of Nottingham, UK) at a flow rate of 60 L.min^−1^. The upper impingement chamber is designed such that at this flow rate through the impinger, the particle cutoff is 6.4 µm and smaller particles pass into the lower impingement. Prior to testing, 7 mL of sterile water was dispensed into the upper chamber and 30 mL to the lower chamber. The experiment consisted in delivering NPs (b-PEI with 2 mg of pGM144 at N/P40) aerosol with the nebulizers (5 mL loaded) into the opening of the GTI until the nebulizer is deactivated. After the experiment, the respirable and non-respirable doses were collected and stored at +4 °C prior to NPs content analyses.

### 2.12. NPs content analyses

To evaluate the amount of encapsulated pDNA inside NPs, 2 µL of a dextran sulfate solution (20 mg/mL) was added to 10 µL of NPs samples before and after nebulization to release pGM144. After 30 min of incubation at room temperature, free pDNA was then quantified using Quant-IT^TM^ PicoGreen^TM^ (ThermoFisher Scientific) following manufacturer protocol in a 96-well black plate. Firstly, 90 µL of TE buffer was added in NPs sample followed by 100 µL of working solution. After 5 min of incubation, fluorescence was measured (480–520 nm) with a multimode microplate reader (Mithras^2^ LB943, Berthold). A standard curve of DNA was realized to determine pDNA concentration. This technique was used to determine the mass distribution and respirable dose for NGI and GTI experiments.

### 2.13. Statistical analyses

All data were analyzed using GraphPad Prism 9.0.2. To assess statistical difference, an unpaired Student *t*-test or Mann–Whitney test was performed. Results were presented as the mean of at least triplicate or more measurements and were considered statistically significant for *p*-value < 0.05 (****cor­responding to a *p*-value < 0.0001, ***corresponding to a *p*-value < 0.001, **corresponding to a *p*-value < 0.01 and *corresponding to a *p*-value < 0.05).

## 3. Results

### 3.1. NPs characterization and pDNA integrity before and after nebulization

**Figure 1.**
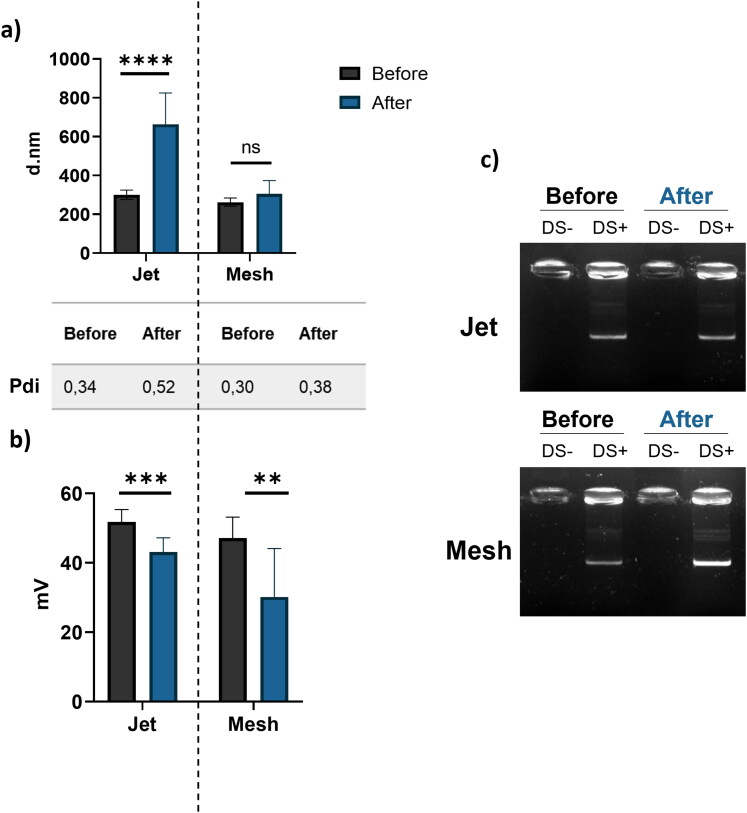
NPs Characterization before and after nebulization; a) Z-average (d.nm) and polydispersity index (PDI) of NPs (250 µg pDNA, N/P 40) before and after nebulization using jet or mesh nebulizer. Three different measurements of three different samples were performed and results are represented as mean ± SD; b) Zeta potential (mV) of NPs (250 µg pDNA, N/P 40) before and after nebulization using jet or mesh nebulizer. Three different measurements of three different samples were performed and results are represented as mean ± SD; c) Electrophorese of b-PEI complex before and after nebulization using jet or mesh nebulizer. To release pGM144, experimentations were performed in presence (+ds) or absence (−ds) of dextran sulfate. For all graphical representations, a Student *t*-test was performed, **** corresponding to a *p* value < 0.0001, *** corresponding to a *p* value < 0.001, ** corresponding to a *p* value < 0.01 and ns corresponding to non-significant.

To appreciate the impact of nebulizer on NPs based b-PEI, different tests have been conducted. First, the hydrodynamic diameter, the polydispersity index (PDI) and the zeta potential were assessed. To conduct these experiments, NPs were prepared and characterized with 250 µg of pGM144 at N/P 40. The Z-average of NPs before aerosol is 281.2 ± 21.7 d.nm with a PDI at 0.32 ± 0.03. These results demonstrate that NPs form a homogeneous population before aerosol. However, the PDI tends to increase after nebulization as shown in Figure 1(a). In fact, after aerosol with jet nebulizer the PDI increased to 0.52 ± 0.02 but when using the mesh nebulizer, PDI remained stable at 0.38 ± 0.03. The same observation can be made with the hydrodynamic diameter, after nebulization using jet nebulizer, it increases up to 662.98 ± 128.74 d.nm while no significant evolution is noticed after nebulization with the mesh nebulizer (304.78 ± 55.14 d.nm) (Figure 1(a)). However, for both nebulizers the zeta potential decreases from 49.42 ± 4.45 mV to 43.10 ± 2.73 mV or 30.12 ± 11.95 mV for jet and mesh nebulizers respectively (Figure 1(b)). Furthermore, to appreciate the impact on pDNA cargo, electrophoresis assays were also conducted. A solution of dextran sulfate was used to release pDNA from b-PEI to observe its integrity after nebulization. The results point out that both devices maintain pDNA integrity as no smear is detected after migration on the agarose gel (Figure 1(c)).

### 3.2. NTA before and after nebulization

**Figure 2.**
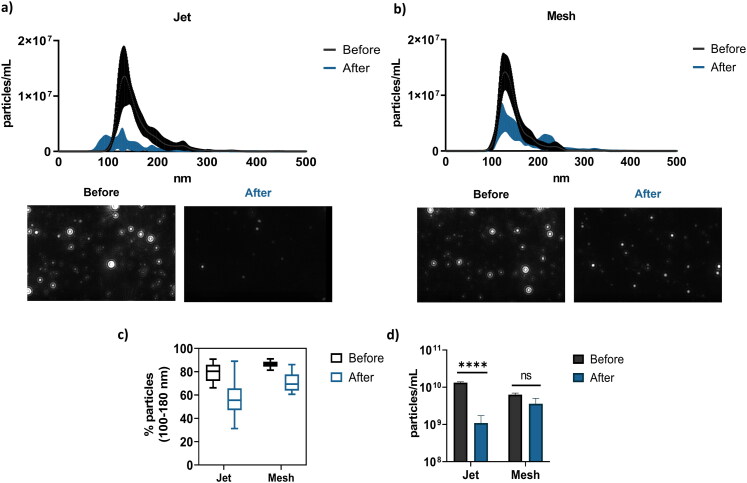
NPs concentration before and after nebulization; a) concentration (particles/mL) as a function of NPs size (nm) before and after nebulization using jet nebulizer. Five different measurements of three different samples were performed and results are represented as mean ± SD; b) Concentration (particles/mL) as a function of NPs size (nm) before and after nebulization using mesh nebulizer. Five different measurements of three different samples were performed and results are represented as mean ± SD; c) Percentage of NPs between 100 to 180 nm before and after mesh nebulization; d) concentration of NPs before and after nebulization using jet or mesh nebulizer. Results are expressed in particles/mL. For all graphical representations, a Student *t*-test was performed, **** corresponding to a *p* value < 0.0001 and ns corresponding to non-significant.

Then, NPs tracking analyses (NTA) were performed using NS300 (Malvern Panalytical) instrument. This technique allows to determine the repartition of NPs population before and after nebulization. These analyses show that before nebulization, 83 ± 5% of total population of NPs containing b-PEI/pGM144 are sized between 100 and 180 nm (Figure 2(c)). After nebulization, this population drops to 57 ± 10% when the aerosol is performed with the PARI LC Plus and to 71 ± 6% with mesh nebulizer. Before nebulization, the particles sized around 140 nm have a concentration comprised between 1 and 2 × 10^7^ particles/mL while this concentration decreases after aerosolization. In fact, Figures 2(a and b) show the pattern of NPs distribution before and after nebulization. When jet nebulizer is used (Figure 2(a)), NPs population is smaller than before aerosol population and more extensive. For the mesh pattern (Figure 2(b)), even if its concentration slightly decreases, the main population remains around 140 nm. Regarding to the concentration of total NPs after mesh nebulization, it is maintained at 10^10^ particles/mL whereas it drops to 10^9^ particles/mL after jet nebulizer (Figure 2(d)).

### 3.3. Validation of the exposure box

**Figure 3.**
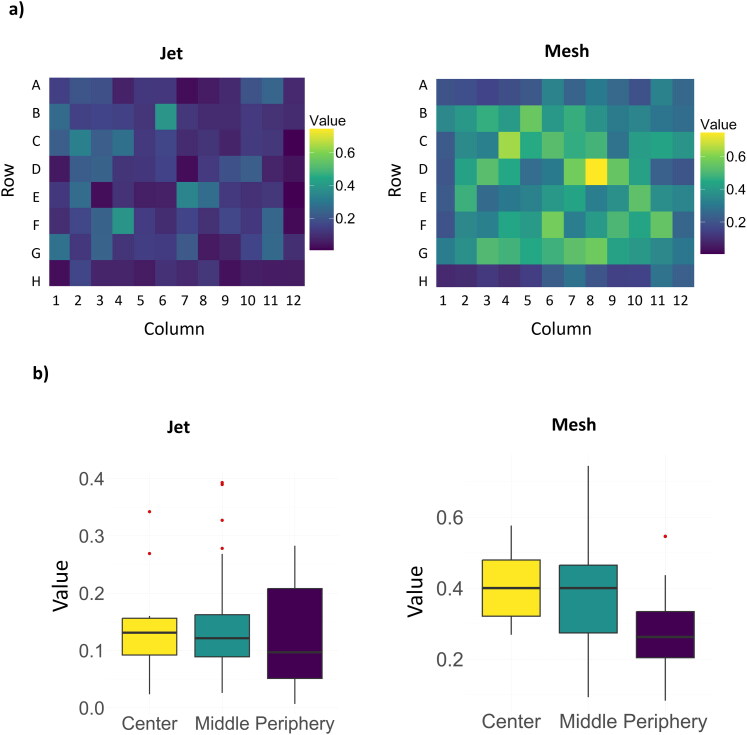
Exposure box validation; a) heat map of the min-max normalized mist repartition inside the exposure box. A549 cells were used to determine this repartition based on luciferase reported gene. Five different nebulization have been performed with b-PEI/pGM144 (250 µg pDNA, N/P 40) using jet or mesh nebulizer to this purpose. The heatmap visualizes the spatial distribution of values across the grid, highlighting patterns of clustering or gradients. The spatial grid was organized as an 8x12 matrix, where each cell corresponds to a physical location on the grid. Values were scaled using a color gradient to enhance interpretability. Brighter colors (yellow) correspond to higher values, while darker colors (blue) correspond to lower values.; b) Boxplot of the distribution of values across three predefined spatial zones: Center, Middle, and Periphery.

To compare the gene delivery efficiency of b-PEI after aerosol using jet or mesh nebulizers, the pyramidal exposure system for *in vitro* experiments must be properly validated to limit any measurement bias. Therefore, the pyramidal 3D-printed (Figure S1) exposure box was tested to determine the homogeneity of the aerosol mist inside it. Different models with various sizes were tested (data not shown) and the best compromise has been settled at 150 mm high for a total volume of ∼1.1 m^3^. A solution of b-PEI/pGM144 (N/P 40) was nebulized either with the mesh or the jet nebulizer onto A549 cells plated in a 96-well plate and the transfection efficiency was measured 24 h after exposition. Each well was then expressed in RLU/mg of proteins and the experiment was repeated 5 times. After normalization, the results were represented as a heat map (Figure 3(a)). The heatmap highlights regions of high values (yellow) and lower-value regions (blue). The closer the value is to 0, the lower is the corresponding reported transfection efficiency. However, more the value is close to 1 more transfection efficiency is high. The normalized data demonstrate that after jet nebulizer, the values range from 0.00 to 0.39 meaning that the mist is evenly distributed. Most of the values are closer to the lowest rather than the highest. However, for mesh nebulizer the values are comprised between 0.06 to 0.78 with less homogeneity. Indeed, the highest values are situated in the middle of the plate suggesting that the mist has the tendency to directly depose in the center and not diffuse like jet nebulizer does. The boxplot confirms this observation and shows that values in the Center zone have higher medians and narrower ranges compared to the Intermediate and Periphery zones (Figure 3(b)). This suggests that values systematically decrease as we move away from the center. For jet nebulizer, this phenomenon is, however, less present.

### 3.4. Transfection efficiency and cell viability

**Figure 4.**
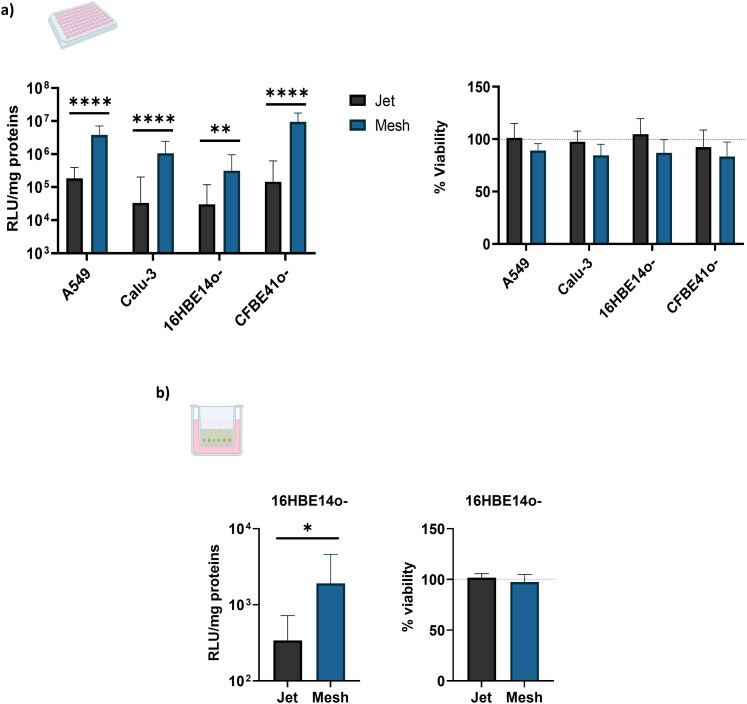
Transfection efficiency and cell viability; a) Luciferase expression and cell viability 24h after transfection on four different submerged cell lines (A549, 16HBE14o-, CFBE41o-, Calu-3) after nebulization using jet or mesh nebulizer of a b-PEI complex solution. Luciferase activity is expressed in relative light unit per milligram of totals proteins (RLU/mg of proteins) and cell viabilities are expressed as a percentage of viability reported to non-transfected cells. Three different nebulization per cells line have been performed and results are represented as mean ± SD. A Student *t*-test was performed to compare the both nebulizers, **** corresponding to a *p* value < 0.0001 and ** corresponding to a *p* value < 0.01; b) Luciferase expression and cell viability 24 h after transfection on 16HBE14o- cell cultivated under air/liquid interface after nebulization using jet or mesh nebulizer of a b-PEI complex solution. Three different nebulization have been performed and 4 transwells have been used by aerosol. Luciferase activity is expressed in relative light unit per milligram of totals proteins (RLU/mg of proteins) and cell viabilities are expressed as a percentage of viability reported to non-transfected cells. Results are represented as mean ± SD. A Mann-Witney test was performed to compare both nebulizers, * corresponding to a *p* value < 0.05.

After nebulization, it is necessary that NPs keep their transfection efficiency capacity. To compare the effect of the nebulization on NPs, four different pulmonary cells (A549, 16HBE14o-, CFBE41o- and Calu-3) were seeded in a 96-well plate and exposed to nebulization. Twenty-four hours after aerosol, luciferase expression was recorded. In each cell line, a significate difference between PARI LC Plus and PARI eFlow was reported. In fact, the mesh nebulizer appears to lead to higher transfection efficiency than jet nebulizer regardless of the cell line (Figure 4(a)). For instance, the reported expression of luciferase is 65 times higher in CFBE41o- with the mesh nebulizer compared to jet nebulizer. This difference is 31 times for Calu-3, 20 times for A549 and 10 times for 16HBE14o-. Regarding cell viability, significant differences between the jet and mesh nebulizer have been reported (Figure 4(a)). However, NPs toxicity increase with the mesh nebulizer, but this increase is correlated to the gene delivery efficacy.

### 3.5. Transfection efficacy on ALI cultures

To appreciate the real impact of nebulizers on gene delivery efficiency toward epithelial cells, air-liquid interface experiments were also conducted. An immortalized cellular model, 16HBE14o-, was allowed to grow under these conditions and then exposed to an aerosol of a solution of b-PEI/pGM144 (2 mg of pGM144, N/P 40). Results show that 24 h after nebulization exposure, luciferase activity was reported. This activity is 5.6 times more important after mesh nebulizer compared to jet nebulizer (Figure 4(b)). In the regard of cell viability, no toxicity was reported with either nebulizer (Figure 4(b)).

### 3.6. In vivo comparison

**Figure 5.**
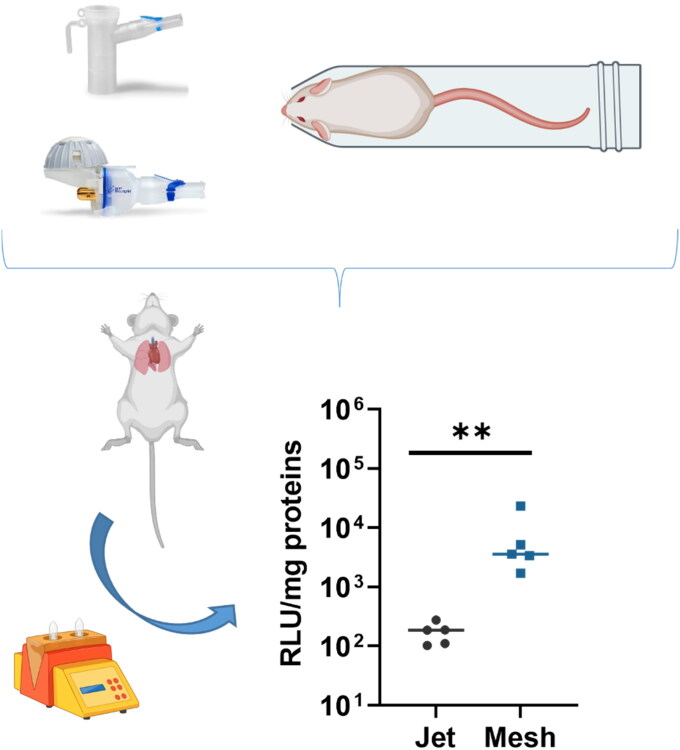
Luciferase expression of lung crushed of mice previously exposed or not to an aerosol of b-PEI complex solution using jet or mesh nebulizer. Results are expressed in relative light unit per milligram of totals proteins (RLU/mg of protein). Five different mice per condition have been used and results are represented as mean ± SD. A Mann-Witney test was performed to compare both nebulizer ** corresponding to a *p* value < 0.01.

Furthermore, to better assess the difference between jet and mesh nebulizers, *in vivo* nebulization was conducted *via* nose-only exposure system. Mice were restrained and exposed to 5 mL of b-PEI/pGM144 solution (2 mg of pGM144, N/P 40) for no more than 28 min. After exposition, mice were monitored daily and euthanized 1 week after aerosolization to collect the trachea and lungs. Results show that after aerosolization using jet nebulizer, the luminescence signal in mice lungs were 1.72 × 10^2^ RLU/mg of proteins whereas it was 7.39 × 10^3^ RLU/mg of protein with mesh nebulizer (Figure 5). This observation is in line with previous results which demonstrate the superiority of the mesh nebulizer over the jet nebulizer for gene transfer using b-PEI.

### 3.7. Aerodynamic features

**Figure 6.**
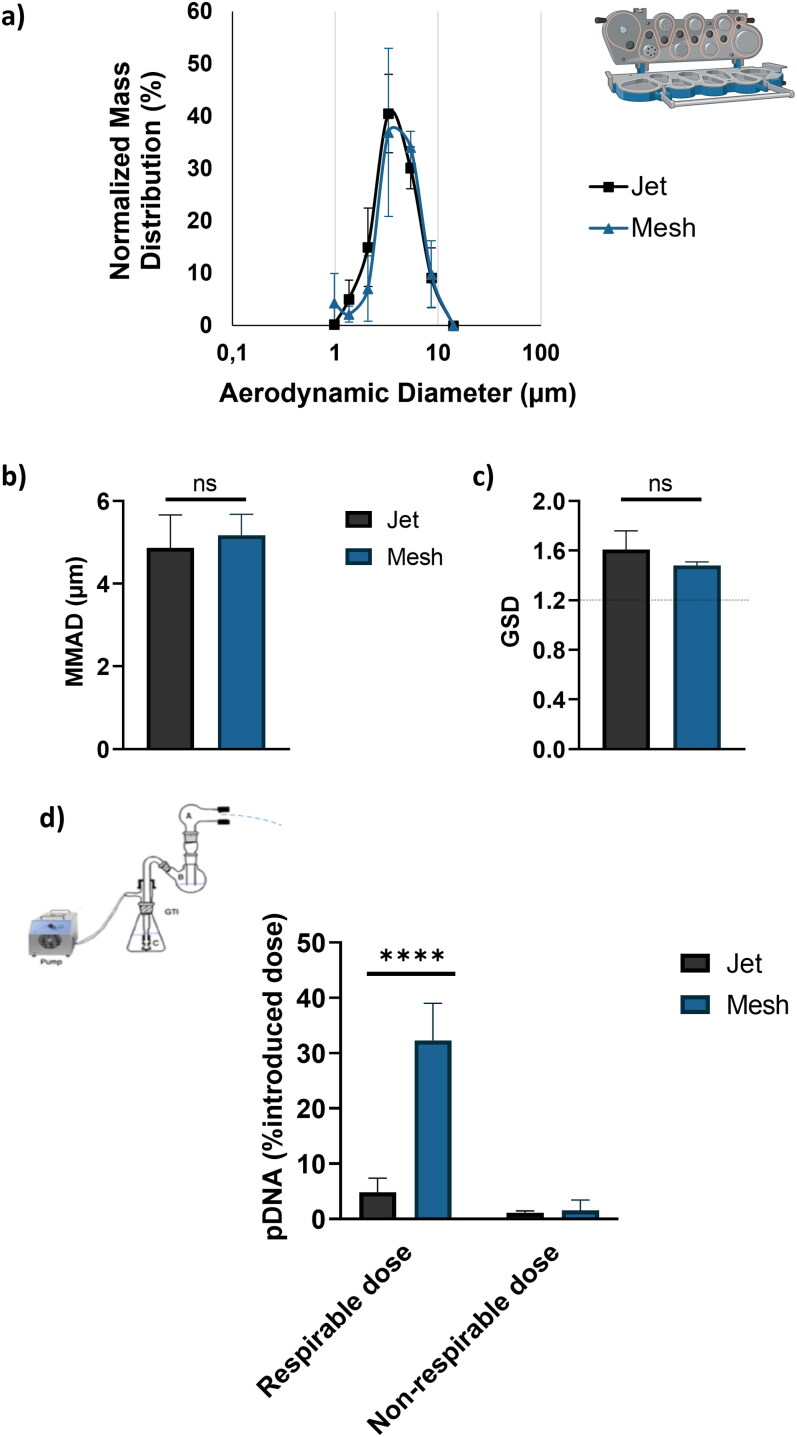
a) Normalized mass distribution as a function of aerodynamic diameter generated using jet or mesh nebulizer. Three nebulizations were performed per nebulizer; b) Median mass aerodynamic diameter (MMAD, µm) and c) geometric standard deviation (GSD) calculated based on the normalized mass distribution; d) comparison of inhalable and non-inhalable dose between jet and mesh nebulizer. The result is expressed in the percentage of introduced pDNA dose. Three nebulizations were performed per nebulizer and a Student *t*-test was performed to compare both nebulizer, **** corresponding to a *p* value < 0.0001, ns corresponding to non-significant.

To investigate the aerodynamic features, cascade impactor experiments were performed. First, the NGI was used to determine the droplet size distribution of aerosol in term of an active ingredient (pDNA) mass distribution (Figure 6(a)). A NPs solution (2 mg pGM144, N/P 40) was nebulized with either jet or mesh nebulizer in the NGI apparatus. The results show that the mass distribution of pDNA into the airborne droplets is similar for both jet and mesh nebulizers. The MMAD and GSD were calculated. No difference was found between the jet and mesh nebulizers. Specifically, the MMAD for the jet nebulizer was 4.86 ± 0.56 µm, and for the mesh nebulizer, it was 5.18 ± 0.4 µm (Figure 6(b)). The GSD was 1.61 ± 0.11 for the jet nebulizer and 1.49 ± 0.02 for the mesh nebulizer. These values are higher than 1.22, indicating that the droplet size distribution is polydisperse (Figure 6(c)). Regarding the respirable dose, GTI experiments were also conducted. The threshold between respirable dose and non-respirable dose was set at 6.4 µm. The results are expressed as a percentage of the introduced pDNA dose and not in terms of NPs. The Figure 6(d) highlights that the respirable dose is 20 times higher when using the mesh nebulizer compared to the jet nebulizer, which explains the observed difference in gene transfection efficacy.

## 4. Discussion

Drug delivery *via* nebulization is a promising noninvasive approach to deliver different kinds of drugs to the lung. However, formulations are required to protect the therapeutic drug and to ensure its delivery into targeted cells. Therefore, this study aims to compare the gene transfection efficacy of two commonly used nebulizers, jet (PARI LC Plus) and mesh (PARI eFlow) nebulizers. Indeed, these nebulizers are based on two different physical principles. The jet nebulizer is based on the Venturi effect, whereas the mesh nebulizer uses a vibrating mesh to generate the aerosol. Each one must be properly investigated to define the best nebulizer for gene delivery applications depending on the formulation. Multiple studies have already compared different types of nebulizers to determine the most suitable for the administrated inhalable drug (Dennis et al., 1990; Kleemann et al., 2004; Arulmuthu et al., 2007; Lenney et al., 2011; Ari, 2014; Park et al., 2021). However, to our knowledge, this comparison has not yet been conducted in terms of gene transfection efficiency with NPs containing pDNA. Therefore, this study was conducted using the well-known cationic polymer, branched polyethyleneimine (b-PEI 25 kDa), and a luciferase CpG-free plasmid. The results demonstrate that both jet and mesh nebulizers can deliver airborne b-PEI-complexed genes to lung epithelial cells in *in vitro* and *in vivo* models. However, these results point out the differences in terms of transfection efficiency between the two nebulizers. Regarding the homogeneity of the mist in the exposure box, the mesh nebulizer seems to produce a more heterogeneous mist compared to the jet nebulizer. This observation could be directly attributed to the design of the exposure box, which may not allow the correct distribution of the mist produced by the mesh nebulizer. It would have been interesting to investigate a larger exposure box and make a comparison with a commercially available system such as Vitrocell^®^ cloud. Furthermore, the mesh nebulizer exhibits higher transfection efficiency compared to the jet nebulizer, as evidence by the increase in luciferase activity. This difference in efficiency may be attributed to the NPs size and concentration, which remain more stable after using the mesh nebulizer rather than the jet nebulizer. In fact, the drop in NPs concentration after jet nebulization represents the main reason for the lower transfection efficiency *in vitro* and *in vivo* compared to the mesh system. However, the size distribution of the droplets produced by the mesh nebulizer and the jet nebulizer is quite similar, so no difference is expected regarding the anatomical targets of regional aerosol deposition in the airways. Based on the total pDNA dosage deposited in the different stages of the NGI cascade impactor, the NPs are evenly distributed in the different droplet sizes generated by the nebulizers. The difference in terms of aerosol deposition is essentially due to the respirable dose of the active ingredient, which is significantly higher with the mesh nebulizer compared to the jet nebulizer. This higher respirable dose increases the NPs concentration into the lung and therefore the gene transfection efficiency. The loose of NPs using jet nebulizer might be explained in part by the remaining volume inside the nebulizer. Indeed, after nebulization, the dead volume is about 160 µL for the jet nebulizer, while no dead volume is found using the mesh nebulizer (Table S1). Another explanation for the NPs concentration difference can be linked to the physical mechanism behind the jet nebulizer. In fact, the Venturi effect is probably more damaging to NPs even if no pDNA degradation is observed. Furthermore, the jet nebulizer used is an older jet and more recent like the PARI LC Sprint^®^ have been developed. However, the physical mechanism to generate the aerosol remains essentially the same, suggesting that similar observations could been made regarding NPs concentration. Moreover, this study was only conducted with a cationic polymer and not a cationic lipid. This kind of vector also represents a great interest in gene delivery by aerosol, as shown by the clinical trial conducted in 2015 by Alton et al. using the GL67A lipid formulation (Alton et al., 2015). During this trial, nebulization was conducted with a jet nebulizer and demonstrated a slight improvement in lung function compared to the placebo in CF patients. In this context, it would have been interesting to perform studies to determine the impact of jet and mesh nebulizers on this same NPs formulation to define the best nebulizer and to fine-tune the formulation. Furthermore, the selection of GL67A for clinical trial was made over b-PEI based on mRNA and protein expression (McLachlan et al., 2011). This comparison was performed using jet nebulizer. A different outcome might have been obtained if a mesh nebulizer was used. Therefore, these results have important implications for the development of nebulized gene therapy strategies for respiratory diseases. The superior performance of the mesh nebulizer in gene delivery using b-PEI suggests its potential utility in clinical settings for the treatment of lung disorders such as cystic fibrosis, asthma, and chronic obstructive pulmonary disease. Future studies should focus on optimizing nebulizer parameters in association with NPs formulation to further enhance gene delivery efficiency and therapeutic efficacy.

## 5. Conclusion

The administration of genes *via* aerosol has been of growing interest since the emergence of lung diseases or pulmonary infections such as SARS-CoV2. To better fine-tune NPs and efficiently target the lung epithelium, it is crucial to understand the potential advantages and limitations of different nebulizers for gene delivery. In summary, this study provides valuable insights into the comparative performance of jet and mesh nebulizers for b-PEI gene delivery. While both types of nebulizers can deliver genes to lung epithelial cells, the mesh nebulizer exhibits higher transfection efficiency *in vitro* and *in vivo* compared to the jet nebulizer. These results underlined the importance of selecting an appropriate nebulizer type based on the NPs formulation to optimize gene delivery outcomes for potential gene therapy applications in the lung.

## Supplementary Material

Supplementary data V2.docx

## Data Availability

The data that support the findings of this study are available on request from the corresponding author.
