# Supplementary material for: Impact of nebulizers on nanoparticles-based gene delivery efficiency: in vitro and in vivo comparison of jet and mesh nebulizers using branched-polyethyleneimine
Source: Drug Deliv. 2025 Feb 10;32(1):2463428. doi: 10.1080/10717544.2025.2463428 (PMC11816613; doi:10.1080/10717544.2025.2463428)
Supplement: Supplementary data V2.docx [file IDRD_A_2463428_SM8436.docx]

**Supplementary data**

**
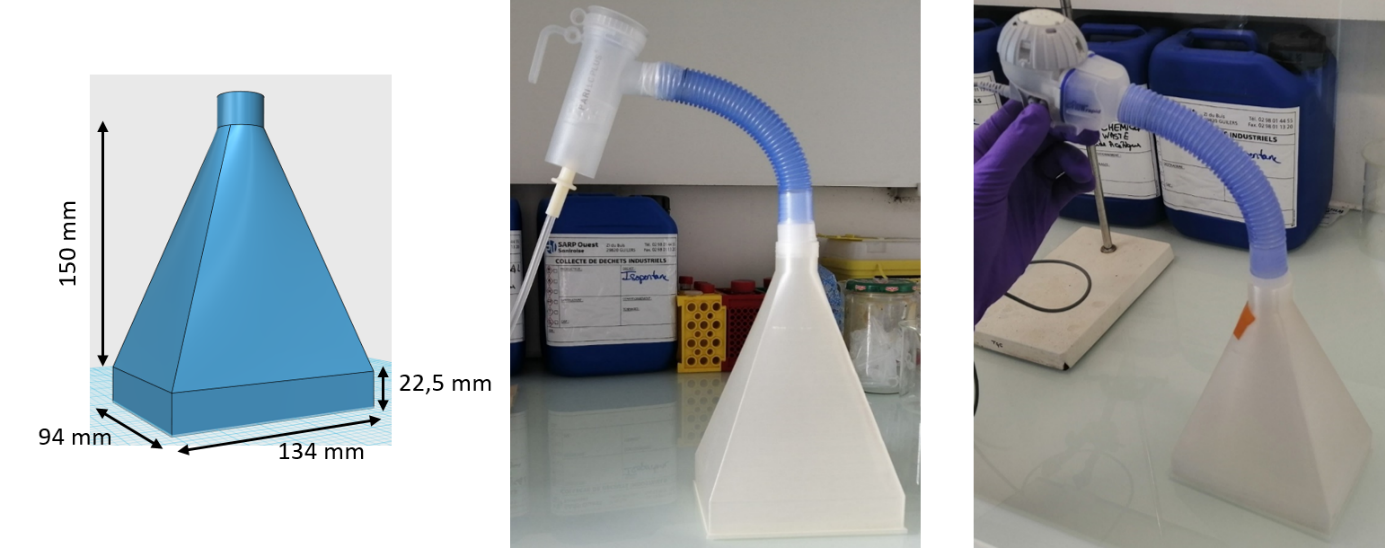
**

**Figure S1:** Design of the pyramidal exposure box. Mesh or jet nebulizers were connected to the exposure box *via* a pipe. The volume of pyramidal box represents approximately 1.1 m^3^.

**
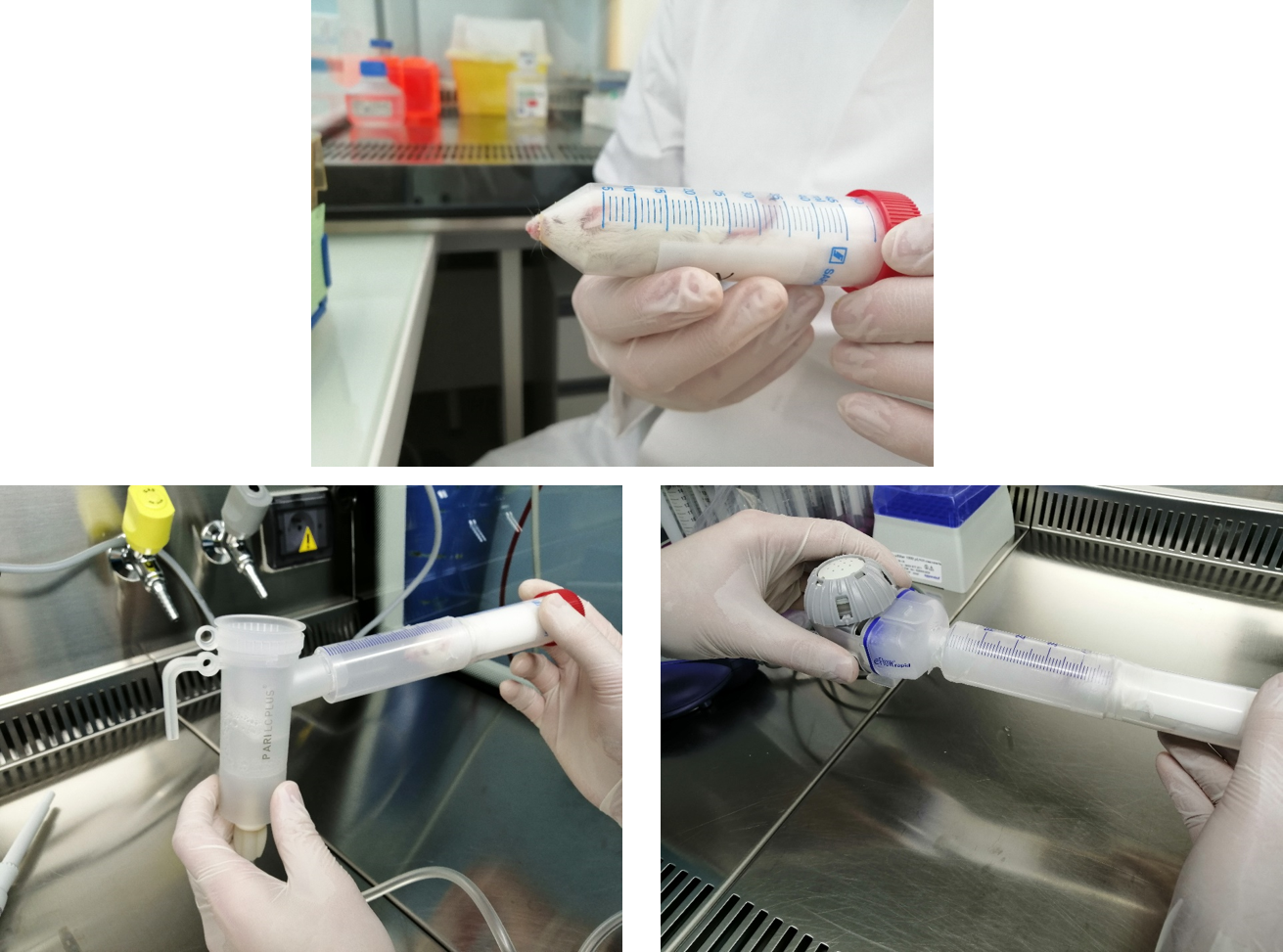
**

**Figure S2:** *In vivo* experiments conducted on Balb/cAnNRj mice using contention tube with an opening for the snout. An inhalation chamber was used to connect the nebulizers to the contention tube.

**Table S1:** Characteristics of the nebulisation performed with 1) jet nebulizer or 2) mesh nebulizer during NGI and GTI experiments. T represents the temperature of the experiment and H the hygrometry.

1. **Jet nebulizer**

|  |  | T (°C) | H (%) | Debit (L/min) | Nebulization time | Death volume (µL) |
| --- | --- | --- | --- | --- | --- | --- |
| Granulometry  (NGI) | n=1 | 20.4 | 30 | 15.5 | 13 min 42 s | 100 |
|  | n=2 |  |  | 15.5 | 12 min 42 s | 150 |
|  | n=3 |  |  | 15.5 | 10 min 48 s | 250 |
| Dosimetry  (GTI) | n=1 | 20.4 | 30 | 60.7 | 12 min 21 s | 50 |
|  | n=2 |  |  | 60.1 | 10 min 41 s | 60 |
|  | n=3 |  |  | 60.0 | 18 min 59 s | 90 |

1. **Mesh nebulizer**

|  |  | T (°C) | H (%) | Debit (L/min) | Nebulization time | Death volume (µL) |
| --- | --- | --- | --- | --- | --- | --- |
| Granulometry  (NGI) | n=1 | 24.7 | 32 | 15.7 | 9 min 36 s | - |
|  | n=2 |  |  | 15.5 | 13 min 40 s | - |
|  | n= |  |  | 15.1 | 11 min 50 s | - |
| Dosimetry  (GTI) | n=1 |  |  | 60.5 | 20 min 19 s | - |
|  | n=2 |  |  | 60.0 | 10 min 06 s | - |
|  | n=3 |  |  | 60.5 | 11 min 24 s | - |
